# Machine Learning Approach for Frailty Detection in Long-Term Care Using Accelerometer-Measured Gait and Daily Physical Activity: Model Development and Validation Study

**DOI:** 10.2196/77140

**Published:** 2025-09-15

**Authors:** Xiaoping Zheng, Ziwei Zeng, Kimberley S van Schooten, Yijian Yang

**Affiliations:** 1 Department of Sports Science and Physical Education The Chinese University of Hong Kong Hong Kong China (Hong Kong); 2 Neuroscience Research Australia University of New South Wales Sydney Australia; 3 School of Population Health University of New South Wales Sydney Australia; 4 CUHK Jockey Club Institute of Ageing The Chinese University of Hong Kong Hong Kong China (Hong Kong)

**Keywords:** frailty, long-term care, gait, physical activity, machine learning

## Abstract

**Background:**

Frailty affects over 50% of older adults in long-term care (LTC), and early detection is critical due to its potential reversibility. Wearable sensors enable continuous monitoring of gait and physical activity, and machine learning has shown promise in detecting frailty among community-dwelling older adults. However, its applicability in LTC remains underexplored. Furthermore, dynamic gait outcomes (eg, gait stability and symmetry) may offer more sensitive frailty indicators than traditional measures like gait speed, yet their potential remains largely untapped.

**Objective:**

This study aimed to evaluate whether frailty in LTC facilities could be effectively identified using machine learning models trained on gait and daily physical activity data derived from a single accelerometer.

**Methods:**

This study is a cross-sectional secondary analysis of baseline data from a 2-arm cluster randomized controlled trial. Of the 164 individuals initially enrolled, 51 participants (age: mean 85.0, SD 9.0 years; female: n=24, 47.1%) met the inclusion criteria of completing all assessments required for this study and were included in the final analysis. Frailty status was assessed using the fatigue, resistance, ambulation, incontinence, loss of weight, nutritional approach, and help with dressing (FRAIL-NH) scale. Participants completed a 5-meter walking task while wearing a 3D accelerometer. Following this task, the accelerometer was used to record daily physical activity over approximately 1 week. A total of 34 dynamic and spatial-temporal gait outcomes, 3 physical activity variables, and 6 demographic characteristics were extracted. Five conventional machine learning models were trained to classify frailty status using a leave-one-out cross-validation approach. Model performance was evaluated based on accuracy and the area under the receiver operating characteristic curve. To enhance model interpretability, explainable artificial intelligence techniques were used to identify the most influential predictive outcomes.

**Results:**

The extreme gradient boosting model demonstrated the optimal performance with an accuracy of 86.3% and an area under the curve of 0.92. Explainable artificial intelligence analysis revealed that older adults with frailty exhibited more variable, complex, and asymmetric gait patterns, which were characterized by higher stride length variability, increased sample entropy, and a lower gait symmetry score.

**Conclusions:**

Our findings suggest that dynamic gait outcomes may serve as more sensitive indicators of frailty than spatial-temporal gait outcomes (eg, gait speed) in LTC settings, offering valuable insights for enhancing frailty detection and management.

## Introduction

Frailty is associated with adverse health outcomes and increased dependency, severely increasing the risk of early mortality in older adults [1]. In long-term care (LTC) facilities, approximately 52.3% of older adults are affected by frailty [2], posing a significant threat to residents’ well-being and sustainability in health care. Effective and efficient frailty screening methods are urgently needed to mitigate these negative outcomes and the progression of frailty among older adults in LTC facilities.

Currently, there is no internationally recognized gold standard for screening or diagnosing frailty for older adults, especially LTC residents. Over 60 different frailty assessment tools have been developed [3]. Fried’s frailty phenotype (FFP) is widely used among community-dwelling older adults [4], while the fatigue, resistance, ambulation, incontinence, loss of weight, nutritional approach, and help with dressing (FRAIL-NH) scale is specifically designed for nursing home settings [5]. However, these assessments are time-consuming, require trained health care professionals, and are often conducted at a single time point, normally after a concern for declined intrinsic capacity has been raised [6]. Given that the transition from healthy aging to frailty is a dynamic and potentially reversible process [7], continuous monitoring is critical for early interventions, which may effectively prevent the frailty progression [8].

Wearable sensor technology and machine learning have emerged as promising alternatives to traditional frailty assessments [9-12]. Accelerometers can continuously monitor older adults’ gait and daily physical activity, while machine learning has been successfully applied to analyze accelerometer data and detect frailty status in community-dwelling older adults [9-12]. Specifically, self-selected gait speed and moderate-to-vigorous physical activity (MVPA) time derived from accelerometer data are significantly correlated with frailty status in community-dwelling older adults [9-12]. However, it remains unclear whether these findings apply to the LTC population, as these individuals often have various physical and cognitive conditions that may affect their mobility [13]. Additionally, LTC residents tend to be more sedentary due to the constrained nature of their living environments [14]. Furthermore, beyond spatial-temporal gait outcomes, the role of dynamic gait outcomes, such as gait regularity and stability derived from accelerometer signals [13], remains underexplored in identifying older adults with frailty. These dynamic outcomes have demonstrated greater sensitivity in distinguishing between different patient groups, such as individuals with chronic low back pain [15-17], and may also be valuable for frailty prediction among older adults.

Apart from this, the emergence of explainable artificial intelligence techniques has addressed the critical challenge of interpreting complex machine learning models in health care applications. These techniques also support compliance with legal and regulatory requirements and help foster trust between clinicians and patients. Among them, Shapley additive explanations (SHAP) is a widely adopted approach grounded in cooperative game theory [16,18]. SHAP enables researchers and clinicians to understand not only which but also how outcomes such as gait variability contribute to the recognition of frailty. Incorporating SHAP enhances model transparency and strengthens clinical relevance, particularly in high-stakes settings like LTC facilities.

Therefore, this study aimed to evaluate whether gait outcomes and daily physical activity data derived from a single accelerometer could identify older adults with frailty in LTC facilities. We hypothesized that machine learning could accurately identify older adults with frailty. By incorporating SHAP, we further hypothesized that accelerometer-derived dynamic gait outcomes would be more sensitive than spatial-temporal gait outcomes in distinguishing between older adults with or without frailty in LTC facilities.

## Methods

### Study Design

This study is a cross-sectional, secondary analysis of baseline data from a 2-arm cluster randomized controlled trial [19]. Baseline data were collected from 20 LTC facilities in Hong Kong. The inclusion criteria were as follows: (1) aged 65 years or older; (2) able to rise from a chair and stand for at least 20 seconds; (3) confirmed by a physician as medically stable to participate in the study; and (4) able to wear an accelerometer continuously for 5 days to measure daily physical activity. Older adults were excluded if they (1) were unable to comprehend instructions, (2) could not complete the exercise program due to medical conditions, or (3) had legal blindness [19].

### Ethical Considerations

The study adhered to the principles of the World Medical Association Declaration of Helsinki and Good Clinical Practice. All participants provided written informed consent, either personally or through a family member, prior to study enrollment. Ethics approval has been acquired from the Research Ethics Board at the Chinese University of Hong Kong and the Joint CUHK-NTEC Clinical Research Ethics Committee (SBRE-21-0413 and CREC-2022-459) [19].

### Data Collection and Processing

Frailty was assessed using the FRAIL-NH scale, with a total score ranging from 0 to 14 [5]. Participants were categorized into 2 groups based on their total score following the cut-off values suggested in a previous study [20]: nonfrail (0-1) and frail (2-14).

During the study, a single accelerometer (ActiGraph GT9X Link IMU) was used, with a sampling frequency of 100 Hz and a range of ±16 gravitational acceleration units (g). Participants were instructed to walk at a comfortable speed over a 5-meter distance twice and the accelerometer was affixed near the L3 vertebra. From the accelerometer data, 34 gait outcomes were extracted from the data (details are provided in Multimedia Appendix 1). These outcomes represent the pace (gait speed, stride length, stride time, stride frequency, and acceleration root mean square), regularity (stride regularity and gait symmetry score), smoothness (index of harmonicity and harmonic ratio), predictability (sample entropy), and stability (maximal Lyapunov exponent and maximal Lyapunov exponent normalized per stride by time) of gait and have been described previously [15].

Regarding daily physical activity, participants were instructed to wear the accelerometer on the right side of their waist (at the anterior superior iliac spine) for 5 consecutive days, removing it only during bathing or before bed [21]. A valid day required at least 8 hours of wear time, and data from at least 3 valid days were included in the analysis using ActiLife software (version 6.13.4, ActiGraph LLC) [22]. Physical activity intensity was classified using established cut-off points for similar populations: sedentary behavior (0-50 counts/min), light physical activity (LPA; 51-759 counts/min), and MVPA (≥760 counts/min) [22]. Three outcomes, including the percentage of time spent in sedentary behavior, LPA, and MVPA, were extracted from the data.

Six demographic characteristics, specifically age, gender, height, body weight, BMI, and use of mobility aids, were collected. The use of mobility aids was categorized into 3 levels: independent, use of cane, and use of walker.

### Model Development and Interpretation

**Figure 1:**
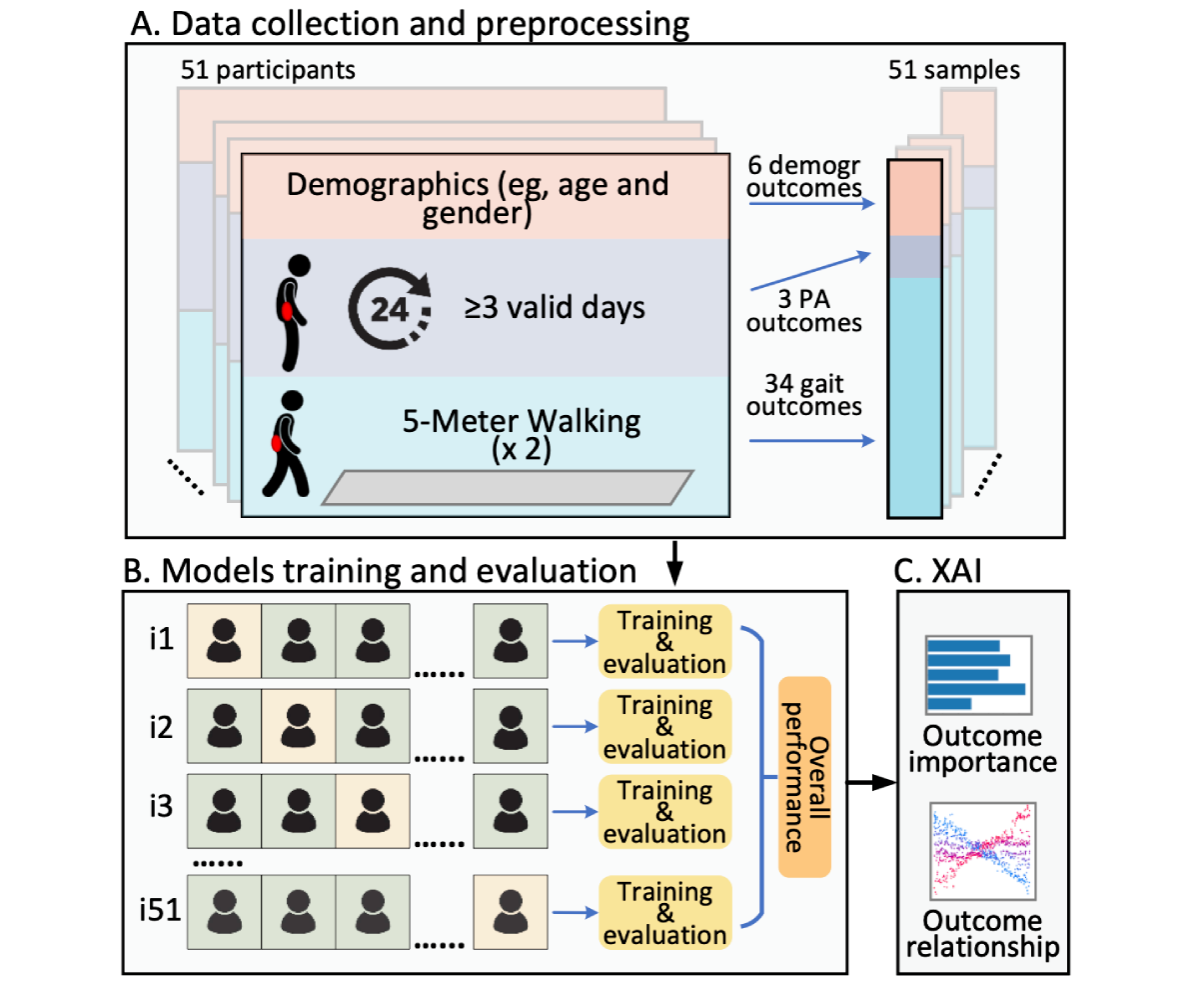
Data processing and analysis. (A) Data collection and preprocessing for demographic, daily physical activity (PA), and gait data. (B) Models training and evaluation based on leave-one-out cross-validation. (C) Explainable artificial intelligence (XAI) for estimating the importance of each outcome and the relationships between different outcomes.

The gait outcomes were averaged across 2 trials, and the daily physical activity outcomes were averaged across valid days. As a result, a total of 43 outcomes (Figure 1A) were used as input to train 5 machine learning classifiers, including extreme gradient boosting (XGBoost), random forest, support vector machine, naive Bayes, and K-nearest neighbors, with labels determined by the FRAIL-NH scale. Detailed descriptions of the corresponding data preprocessing of each model are provided in Multimedia Appendix 2.

Model performance for each classifier was evaluated using leave-one-out cross-validation (LOO-CV), where the data from 1 participant was left out for testing, and the remaining participants were used for training (Figure 1B). This process was repeated for each participant to ensure an unbiased assessment of model performance. For each iteration, a Bayesian optimization approach [23] with 5-fold cross-validation was used to identify the best hyperparameters for the classifier. This process followed a nested cross-validation design [24]. The details of the parameter space and settings of each machine learning model are provided in Multimedia Appendix 3. Model performance was evaluated by aggregating results from LOO-CV to compute the following metrics: accuracy, sensitivity, specificity, area under the receiver operating characteristic curve (AUC), and *F*_1_-score.

To explore the association between gait outcomes, daily physical activity, and frailty, SHAP was used to interpret the results of the best classifier. SHAP estimates the contribution of each outcome to the classification process, as well as the interactions between selected outcomes using principles from game theory (Figure 1C).

In this study, SHAP values were computed for each testing participant during each iteration of the LOO-CV, using the corresponding model trained without that participant. The SHAP values from all iterations were then aggregated, leveraging the additivity property of SHAP [16,18], to generate a global interpretation of feature importance across the entire dataset. This approach ensures faithful, subject-specific explanations while maintaining the validity of the cross-validation framework.

To allow for the verification and reproduction of results, the project repository has been made publicly available [25]. It includes the source code and log files of all experiments.

### Statistical Analysis

Statistical analyses were conducted using SPSS version 27.0 (IBM Corporation) with a significance level set at *P*<.05. Continuous variables were presented as mean (SD) for normally distributed data or median (IQR) for non-normally distributed data. Categorical variables were expressed as frequency and percentage. The normality of continuous variables was assessed using the Shapiro-Wilk test. To compare demographic characteristics between the nonfrail and frail groups, independent samples *t* tests were used for normally distributed variables, while the Mann-Whitney U test was applied for non-normally distributed variables. For categorical variables, the Chi-square test (or Fisher exact test, where appropriate) was used to compare proportions between the 2 groups.

## Results

### Participant Characteristics

Among the 164 participants recruited from 20 LTC facilities, 113 were excluded for the following reasons: aged younger than 65 years, deceased, or withdrew from participation (n=15); unwilling to wear an accelerometer or insufficient accelerometer data for daily activity (n=82); and missing accelerometer data for gait (n=16). This left 51 participants for data analysis, with their demographic characteristics summarized in Table 1. Statistically significant differences between the nonfrail and frail groups were observed in body weight (nonfrail: mean 57.3, SD 10 kg; frail: mean 50.7, SD 9.4 kg; *P*=.02) and the use of walking aids (*P*=.008).

**Table 1:** Demographic characteristics of participants, mean (SD).

Demographic		Nonfrail (n=26)	Frail (n=25)	*P* value	
Age (years), mean (SD)		83.2 (10.6)	87.0 (7.0)	.14	
**Gender, n**					.48
	Female	11	13		
	Male	15	12		
Height (cm), mean (SD)		159.0 (9.7)	157.2 (10.9)	.52	
Body weight (kg), mean (SD)		57.3 (10.0)	50.7 (9.4)	.02	
BMI (kg/m^2^), mean (SD)		22.6 (3.5)	20.7 (4.0)	.07	
**Walking aids, n**					.008
	Independent	16	5		
	Use of cane	9	16		
	Use of walker	1	4		

### Model Performance

**Figure 2:**
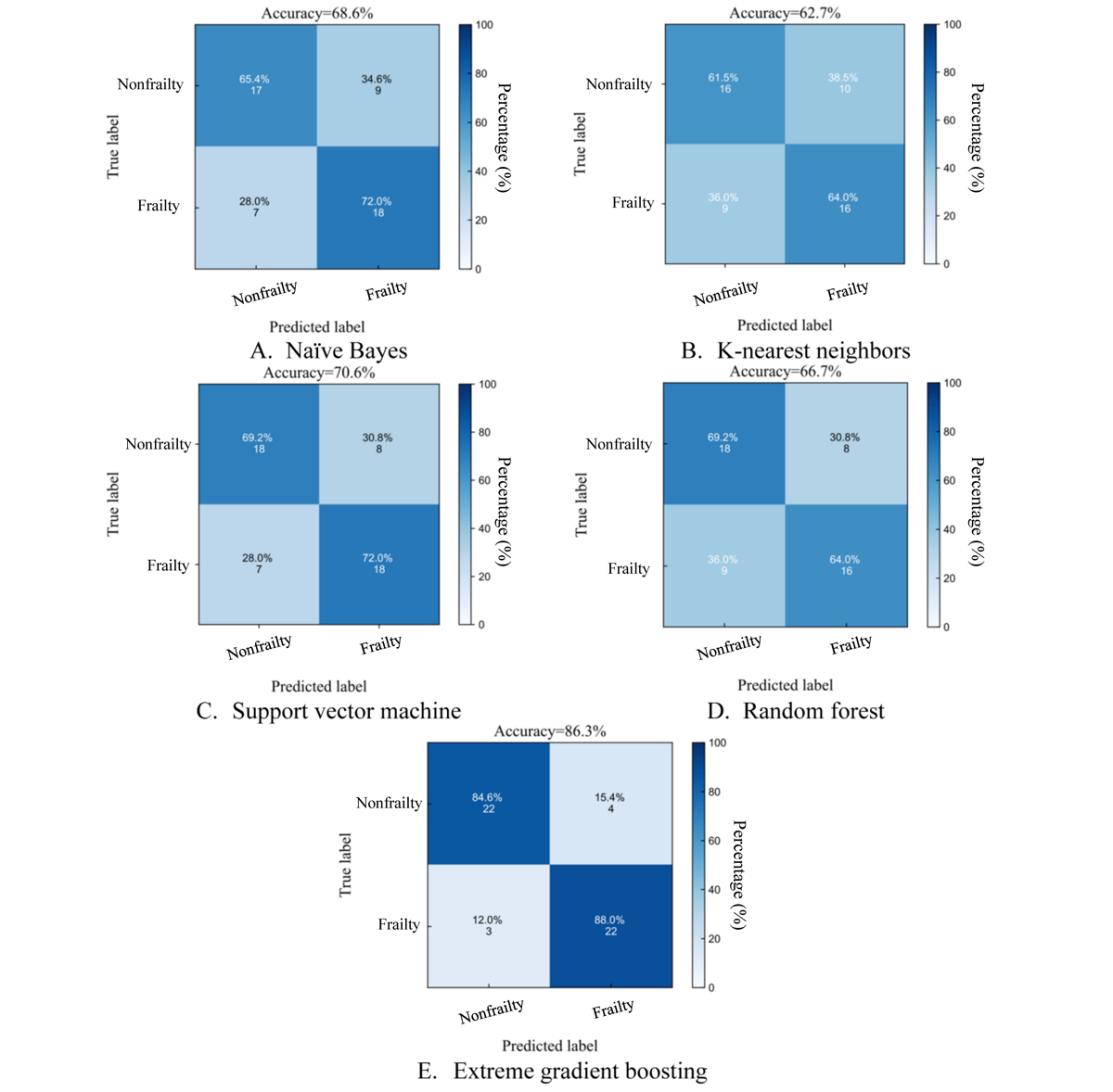
Confusion matrix comparisons between machine learning classifiers.

**Figure 3:**
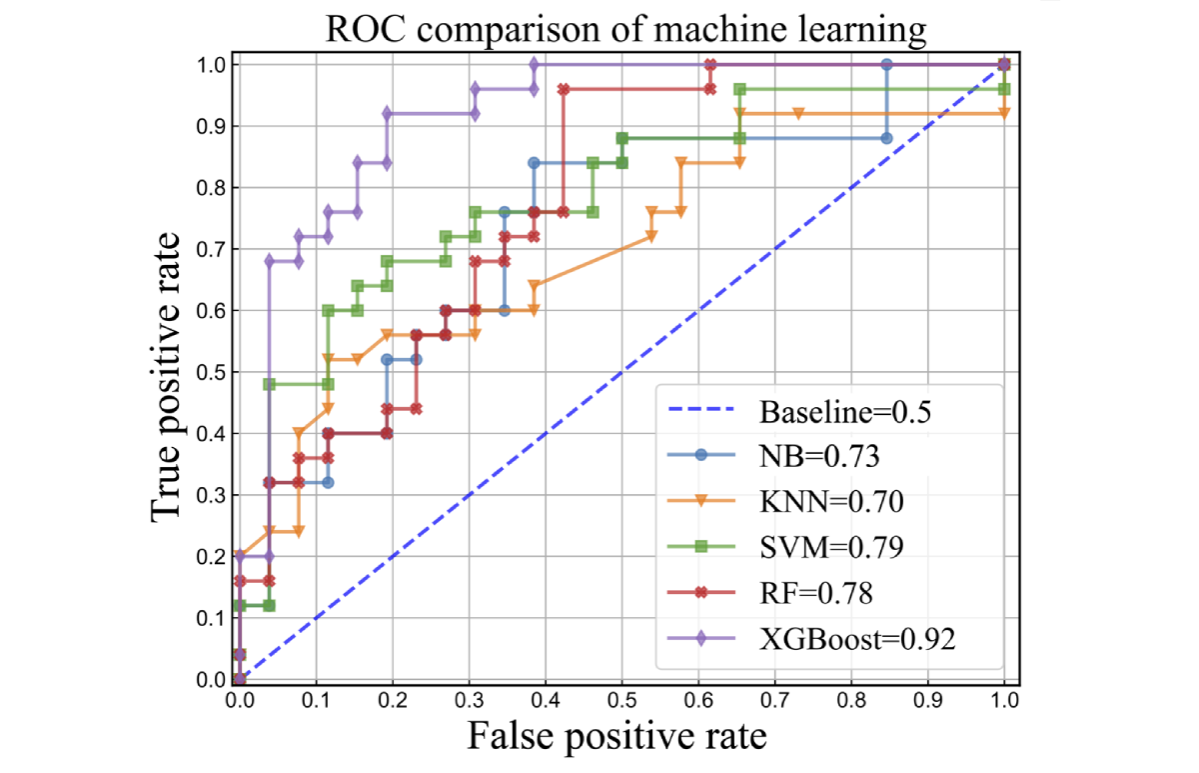
Comparison of the area under the curve between machine learning models. ROC: receiver operating characteristic curve; NB: naive Bayes; KNN: K-nearest neighbors; SVM: support vector machine. RF: random forest; XGBoost: extreme gradient boosting.

Table 2 presents all performance metrics for each model and Figure 2 shows their confusion matrices to offer a detailed view of individual model performance. XGBoost demonstrated the best classification performance compared to other machine learning models, achieving an AUC of 0.92 (Figure 3), with an accuracy of 86.3%, sensitivity of 0.85, specificity of 0.88, and an *F*_1_-score of 0.86.

**Table 2:** Performance metric comparison between machine learning models.

Model	Accuracy (%)	AUC^a^	Sensitivity	Specificity	*F*_1_-score
NB^b^	68.6	0.73	0.65	0.72	0.68
KNN^c^	62.7	0.70	0.62	0.64	0.63
SVM^d^	70.6	0.78	0.69	0.72	0.71
RF^e^	66.7	0.79	0.69	0.64	0.68
XGBoost^f^	86.3	0.92	0.85	0.88	0.86

^a^AUC: area under the receiver operating characteristic curve.

^b^NB: naive Bayes.

^c^KNN: K-nearest neighbors.

^d^SVM: support vector machine.

^e^RF: random forest.

^f^XGBoost: extreme gradient boosting.

SHAP was used to interpret the classification process of XGBoost, as it demonstrated the best performance. The top 10 outcomes based on absolute SHAP values are presented in Multimedia Appendix 4. The elbow plot identified an “elbow” at 5, suggesting that the top 5 outcomes were the most influential in the classification. These key outcomes include stride length variability, sample entropy in the vertical direction, body weight, BMI, and gait symmetry score in the vertical direction.

**Figure 4:**
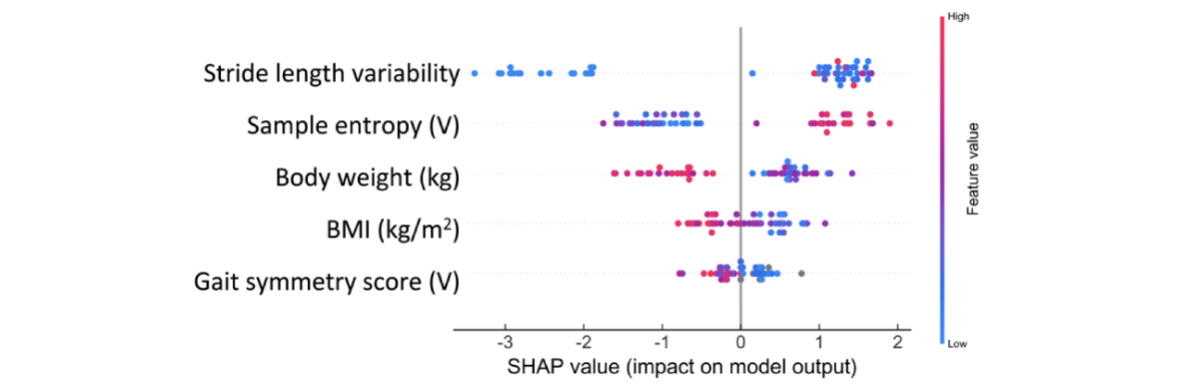
Relationship between Shapley additive explanations (SHAP) value and key outcomes.

Figure 4 illustrates the relationships between these outcomes and their corresponding SHAP values. Negative SHAP values indicate a contribution toward the nonfrail group, while positive values indicate a contribution toward the frail group. Larger absolute SHAP values represent a greater influence on classification. The figure shows that predicted older adults without frailty tend to have lower stride length variability, whereas participants with high sample entropy (greater gait complexity) are more likely to be classified as frail. Similarly, higher body weight and BMI values are associated with the nonfrail group. Higher gait symmetry score (indicating more symmetry; Multimedia Appendix 5) in the vertical direction contributes to the frail group, while lower values are associated with a nonfrailty classification.

**Figure 5:**
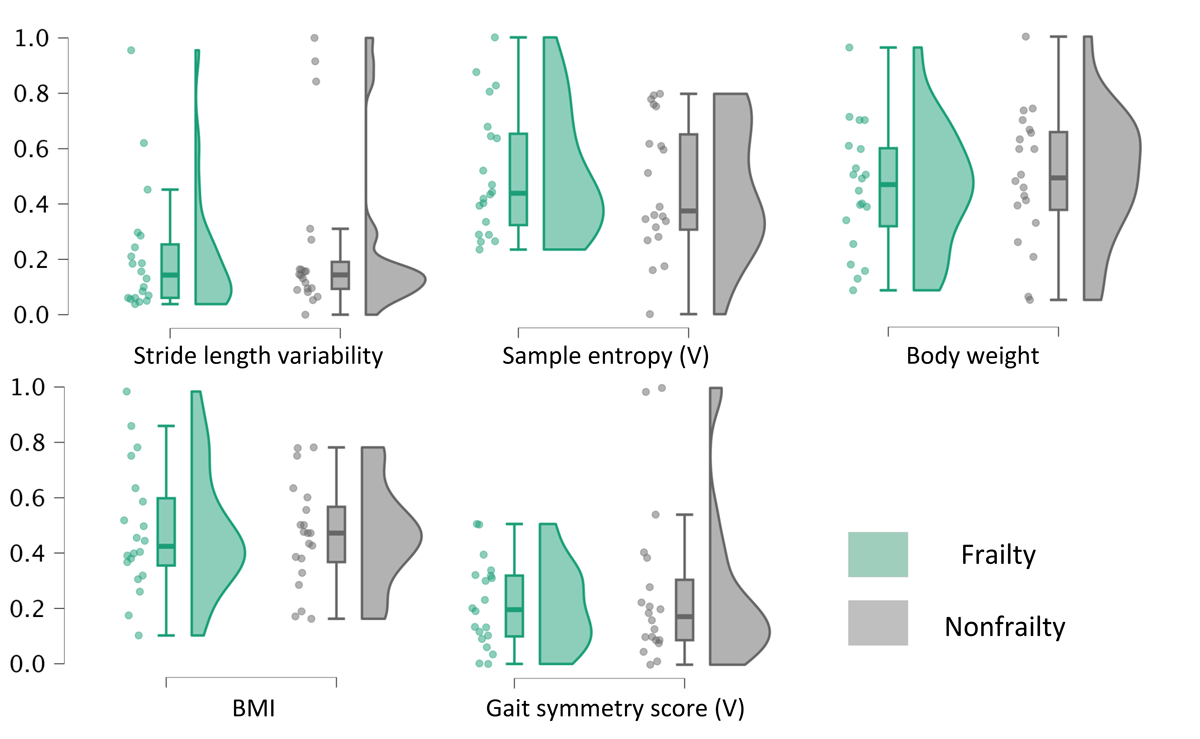
Raincloud plot for the top 5 key outcomes (normalized to a 0-1 scale). The dots represent individual data points; the thick line within each box plot represents the median, while the box boundaries show the interquartile range; the density plot reflects a kernel density estimate, illustrating the distribution of data across different values—wider sections indicate higher data concentration. V: vertical direction.

Figure 5 presents raincloud plots of the 5 key outcomes. The raincloud plot combines a kernel density plot with a box plot, with dots representing individual data points. All values were normalized to a 0-1 scale for comparability. Compared with the nonfrail group, the frail group exhibited higher stride length variability, greater sample entropy in the vertical direction, and decreased gait symmetry score in the vertical direction.

## Discussion

### Principal Findings

To the best of our knowledge, this study is among the first investigations that used machine learning in conjunction with accelerometer-derived gait and daily physical activity data to detect frailty among older adults in LTC facilities. Our results confirm that machine learning models, particularly XGBoost, demonstrated accurate classification performance in a LOO-CV (AUC=0.92, accuracy=86.3%). Consistent with our hypothesis, dynamic gait outcomes, including stride length variability, sample entropy (vertical direction), and gait symmetry (vertical direction), are more sensitive in distinguishing frailty status among LTC-dwelling older adults compared with traditional spatial-temporal parameters (eg, gait speed). These biomechanical markers, combined with anthropometric measures (body weight and BMI), emerged as the most influential predictors.

Stride length variability contributed the most to the classification process, with higher variability observed in the frail group. Previous research has reported similar findings, showing that older adults with frailty exhibit greater stride length variability in community settings [26,27]. Larger stride-to-stride fluctuations in stride length may reflect reduced accuracy and reliability of the neuromuscular systems that regulate gait rhythm [28]. Given that increased fall risk [29], age-related physical decline [30], and cognitive impairments are linked to frailty [31], it is not surprising that older adults with frailty exhibit higher gait variability [32-34]. Sample entropy was used to quantify gait predictability and complexity [35]. Higher entropy values (less predictability) in the frail group may suggest disrupted locomotor control, likely tied to neurological or neuromuscular dysfunction [36-38]. This aligns with findings that gait irregularity discriminates older adults with prefrailty from those without frailty [39] in community-dwelling settings and correlates with pathologies such as Parkinson disease and high fall risk [38]. Gait symmetry, which provides insight into the consistency between 2 consecutive steps, was lower in older adults with frailty, indicating greater degrees of asymmetry. Left-right gait coordination is primarily governed by localized neuronal networks, or central pattern generators, as observed in animal studies [40]. In healthy individuals, this coordination occurs automatically with limited cognitive input [41]. However, with aging, sarcopenia, or cognitive impairment, gait becomes less automatic and left-right coordination may increasingly depend on attentional demands, particularly in individuals with frailty [41,42]. Additionally, studies have shown no strong association between gait variability and asymmetry, suggesting that asymmetry represents an independent measure of gait impairment linked to distinct pathological causes [41]. Collectively, these findings suggest that older adults with frailty in LTC facilities exhibit more variable, irregular, and asymmetric gait patterns compared to those without frailty.

The frail group had significantly lower body weight compared with the nonfrail group, which aligns with sarcopenia’s role in frailty pathogenesis, where muscle mass loss contributes to both weight reduction and functional decline [43]. Additionally, the lower BMI may reflect nutritional deficits commonly seen in older adults residing in LTC facilities, further exacerbating frailty progression [44]. These findings highlight the value of integrating anthropometric data with dynamic gait metrics for comprehensive frailty screening in LTC settings.

Notably, dynamic gait outcomes contributed more to the classification process than gait speed. In community-dwelling older adults, self-selected gait speed has been widely recognized as a key indicator for distinguishing frailty from nonfrailty [11,12] and has been incorporated into screening tools like the FFP [45]. However, in this study, the low SHAP value of gait speed indicates its limited contribution to the classification process among older adults in LTC facilities. One possible explanation is that self-selected gait speed is influenced by multiple factors beyond walking capacity. For instance, in this study, 21.6% of older adults had experienced at least one fall in the past year. Due to fear of falling or other safety concerns, they may voluntarily reduce their gait speed despite being physically capable of walking faster [46]. Another possible explanation is the environmental constraints of LTC facilities in Hong Kong. The care homes in Hong Kong typically have narrow spaces, and gait measurements were conducted in hallways, which may have influenced gait speed. As a result, gait speed alone may not be a sufficiently sensitive measure for frailty classification in this population and settings.

Previous studies have reported that older adults with frailty tend to have lower levels of daily physical activity [47], and higher physical activity levels have been shown to protect against frailty [48]. In this study, the frail group exhibited a lower percentage of time spent in sedentary behavior (*P*=.001) and a higher percentage of time in LPA (*P*=.001), but no significant difference was observed in MVPA (Multimedia Appendix 6). However, SHAP values indicated that physical activity outcomes contributed minimally to model predictions. This suggests that in LTC settings, the current activity measures, based on time spent percentage, may not strongly differentiate frailty status (Multimedia Appendix 4). One possible reason is that the current physical activity metrics are relatively heterogeneous, covering a broad range of percentage values with considerable variability and outliers. To enhance frailty classification, more advanced measures such as postural transitions (eg, sit-to-stand [49]) and physical activity patterns [50] should be considered to develop a more robust model capable of accurately distinguishing between frail and nonfrail states among this vulnerable population.

Compared with traditional frailty assessment tools, such as the FFP and the short physical performance battery [51,52], the proposed method leverages wearable sensor-derived gait and physical activity outcomes analyzed through machine learning. This approach enables a more objective, continuous, and data-driven assessment of frailty. It also holds promise for the early detection of prefrailty and frailty in a scalable, unobtrusive, and real-time manner across diverse care settings, ultimately supporting more timely and targeted interventions.

### Limitations

The walking task in this study was limited to a 5-meter distance due to space constraints in Hong Kong LTC facilities. While 2.4-, 4.6-, or 6-meter walking tests are commonly used for clinical gait analysis [53] and longer walks likely would have led to fatigue, a longer distance could have provide more reliable estimates of gait characteristics. For instance, sample entropy typically requires at least 10 seconds of signal data for reliable computation. In this study, the average walking speed was 0.46 m/s (SD 0.15 m/s), meaning that the recorded acceleration data exceeded 10 seconds, meeting the requirement for valid sample entropy calculations. Additionally, we tested the stability of sample entropy (Multimedia Appendix 7), which showed that using 70% of the current data length was sufficient to obtain stable results. Therefore, we believe that the sample entropy findings in this study are reliable. Given the limited number of participants in this study, a LOO-CV approach was used to improve the generalizability of the machine learning results and reduce the risk of overfitting. Lastly, the cross-sectional design precludes causal inferences, and the FRAIL-NH scale, though validated for LTC settings [5,20], may not fully capture frailty’s dynamic progression. Longitudinal studies with repeated measures are warranted to validate these findings.

### Conclusions

This study demonstrated that machine learning models, particularly XGBoost, effectively identify frailty in LTC older adults using accelerometer-derived gait and daily physical activity data. Our findings underscore the superiority of dynamic gait outcomes, including stride length variability, sample entropy (vertical direction), and gait symmetry (vertical direction), over traditional spatial-temporal outcomes in distinguishing frailty status. These outcomes reflect nuanced neuromuscular insights into frailty pathophysiology. The complementary role of anthropometric measures (body weight and BMI) further hints toward an interplay between sarcopenia, nutritional status, and functional decline in this population.

## Appendix

Multimedia Appendix 1 The included gait outcomes.

Multimedia Appendix 2 Details of data preprocessing for each model.

Multimedia Appendix 3 Hyperparameter space.

Multimedia Appendix 4 Absolute Shapley additive explanations values for the top 10 outcomes and elbow plot.

Multimedia Appendix 5 Gait symmetry score calculation.

Multimedia Appendix 6 Raincloud plots for the daily physical activity outcomes.

Multimedia Appendix 7 The averaged sample entropy values in the vertical direction over all participants based on different length of acceleration signals.

## Abbreviations

AUC: area under the receiver operating characteristic curve
FRAIL-NH: fatigue, resistance, ambulation, incontinence, loss of weight, nutritional approach, and help with dressing scale
FFP: Fried’s frailty phenotype
LOO-CV: leave-one-out cross-validation
LPA: light physical activity
LTC: long-term care
MVPA: moderate-to-vigorous physical activity
SHAP: Shapley additive explanations
XGBoost: extreme gradient boosting
