# Correction: Machine Learning Approach for Frailty Detection in Long-Term Care Using Accelerometer-Measured Gait and Daily Physical Activity: Model Development and Validation Study

**DOI:** 10.2196/85173

**Published:** 2025-10-29

**Authors:** Xiaoping Zheng, Ziwei Zeng, Kimberley S van Schooten, Yijian Yang

**Affiliations:** 1 Department of Sports Science and Physical Education The Chinese University of Hong Kong Hong Kong China (Hong Kong); 2 Neuroscience Research Australia University of New South Wales Sydney Australia; 3 School of Population Health University of New South Wales Sydney Australia; 4 CUHK Jockey Club Institute of Ageing The Chinese University of Hong Kong Hong Kong China (Hong Kong)

In “Machine Learning Approach for Frailty Detection in Long-Term Care Using Accelerometer-Measured Gait and Daily Physical Activity: Model Development and Validation Study” (JMIR Aging 2025;1(8) e77140) the authors made a few corrections.

In the Results section of the Abstract, the sentence:

Explainable artificial intelligence analysis revealed that older adults with frailty exhibited more variable, complex, and asymmetric gait patterns, which were characterized by higher stride length variability, increased sample entropy, and a higher gait symmetry score.

Has been revised to:

Explainable artificial intelligence analysis revealed that older adults with frailty exhibited more variable, complex, and asymmetric gait patterns, which were characterized by higher stride length variability, increased sample entropy, and a lower gait symmetry score.

In the Results section, the sentence:

Higher gait symmetry score (indicating less symmetry; Multimedia Appendix 5) in the vertical direction contributes to the frail group, while lower values are associated with a nonfrailty classification.

Has been revised to:

Higher gait symmetry score (indicating more symmetry; Multimedia Appendix 5) in the vertical direction contributes to the frail group, while lower values are associated with a nonfrailty classification.

In the Results section, the sentence:

Compared with the nonfrail group, the frail group exhibited higher stride length variability, greater sample entropy in the vertical direction, and increased gait symmetry score in the vertical direction.

Has been revised to:

Compared with the nonfrail group, the frail group exhibited higher stride length variability, greater sample entropy in the vertical direction, and decreased gait symmetry score in the vertical direction.

In the Multimedia Appendix 5, the formula:



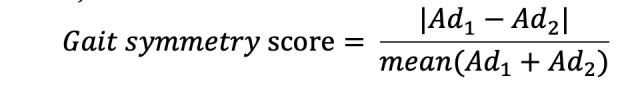



Has been revised to:



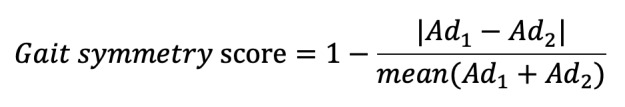



The correction will appear in the online version of the paper on the JMIR Publications website, together with the publication of this correction notice. Because this was made after submission to full-text repositories, the corrected article has also been resubmitted to those repositories.

