# Supplementary material for: Machine Learning Approach for Frailty Detection in Long-Term Care Using Accelerometer-Measured Gait and Daily Physical Activity: Model Development and Validation Study
Source: JMIR Aging. 2025 Sep 15;8:e77140. doi: 10.2196/77140 (PMC12481141; doi:10.2196/77140)
Supplement: Multimedia Appendix 1 [file aging_v8i1e77140_app1.docx]

Multimedia Appendix 1

Supplement Table 1. The Included Gait Outcomes.

| Category | Gait Outcomes |
| --- | --- |
| Pace | Gait Speed |
|  | Stride Length |
|  | Stride Time |
|  | Stride Frequency |
|  | Acceleration Root Mean Square |
| Regularity | Stride Frequency Variability-V |
|  | Stride Frequency Variability-ML |
|  | Stride Frequency Variability-AP |
|  | Gait Speed Variability (Stride) |
|  | Stride Time Variability |
|  | Stride Length Variability |
|  | Stride Regularity-V |
|  | Stride Regularity-ML |
|  | Stride Regularity-AP |
|  | Stride Regularity-All |
|  | Gait Symmetry score |
|  | Gait Symmetry-V |
|  | Gait Symmetry-AP |
| Smoothness | Index Of Harmonicity-V |
|  | Index Of Harmonicity-ML |
|  | Index Of Harmonicity-AP |
|  | Index Of Harmonicity-All |
|  | Harmonic Ratio-V |
|  | Harmonic Ratio-ML |
|  | Harmonic Ratio-AP |
| Predictability | Sample Entropy-V |
|  | Sample Entropy-ML |
|  | Sample Entropy-AP |
| Stability | Maximal Lyapunov Exponent-V |
|  | Maximal Lyapunov Exponent-ML |
|  | Maximal Lyapunov Exponent-AP |
|  | Maximal Lyapunov Exponent Normalized Per Stride by Time-V |
|  | Maximal Lyapunov Exponent Normalized Per Stride by Time-ML |
|  | Maximal Lyapunov Exponent Normalized Per Stride by Time-AP |

AP: Anterior-posterior direction, ML: medio-lateral direction, V: vertical direction.
