# Supplementary material for: Machine Learning Approach for Frailty Detection in Long-Term Care Using Accelerometer-Measured Gait and Daily Physical Activity: Model Development and Validation Study
Source: JMIR Aging. 2025 Sep 15;8:e77140. doi: 10.2196/77140 (PMC12481141; doi:10.2196/77140)
Supplement: Multimedia Appendix 2 [file aging_v8i1e77140_app2.docx]

Multimedia Appendix 2

Supplement Table 2. Details of Data Preprocessing for Each Model.

| Models | Preprocessing |
| --- | --- |
| Naïve Bayes, K-Nearest Neighbors, Support Vector Machine | Missing values were imputed using the mean of all available data. The dataset was then normalized using the min-max scaling method. Subsequently, kernel principal component analysis (kPCA) was applied to reduce the dimensionality of the input features. To retain 90% of the variance, 15 principal components were selected for classifiers training. |
| Random Forest | Missing values were imputed using the mean of all available data. |
| eXtreme Gradient Boosting | No preprocessing was conducted. |
