# Supplementary material for: Machine Learning Approach for Frailty Detection in Long-Term Care Using Accelerometer-Measured Gait and Daily Physical Activity: Model Development and Validation Study
Source: JMIR Aging. 2025 Sep 15;8:e77140. doi: 10.2196/77140 (PMC12481141; doi:10.2196/77140)
Supplement: Multimedia Appendix 3 [file aging_v8i1e77140_app3.docx]

Multimedia Appendix 3

Supplement Table 3. Hyper Parameter Space.

| Model | Hyper parameter space |
| --- | --- |
| Naïve Bayes | The proportion of the largest variance across all features was set between 10^-11^and 10^-7^, with increments of 10^-10^. |
| K-Nearest Neighbors | The number of neighbors was varied from 1 to 25. Different algorithms for computing the nearest neighbors, including “ball_tree,” “kd_tree,” and “brute,” were evaluated. Additionally, the weight functions “uniform” and “distance” were examined. |
| Support Vector Machine | The radial basis function (“rbf”) kernel was utilized. The box constraint parameter (C) was varied from 1 to 250. The polynomial degree was set between 1 and 50. The gamma parameter was adjusted from 0.01 to 10 in increments of 0.05. |
| Random Forest | The number of trees was varied from 10 to 1000 in increments of 10. The hyperparameters for individual trees were set as follows: the maximum tree depth was varied from 1 to 15, the maximum number of leaf nodes was adjusted from 5 to 20, and the minimum number of samples required at a leaf node was varied between 5 and 20. |
| eXtreme Gradient Boosting | The learning rate (eta) was varied logarithmically from 0.01 to 1. The booster types “gbtree” and “dart” were explored. The maximum tree depth was adjusted between 1 and 15. The number of estimators (trees) was varied from 100 to 1000. |

For each classifier, 15 parameter combination trails were searched, and a Bayesian optimization approach determined the best hyperparameters for each classifier.
