# Supplementary material for: Machine Learning Approach for Frailty Detection in Long-Term Care Using Accelerometer-Measured Gait and Daily Physical Activity: Model Development and Validation Study
Source: JMIR Aging. 2025 Sep 15;8:e77140. doi: 10.2196/77140 (PMC12481141; doi:10.2196/77140)
Supplement: Multimedia Appendix 4 [file aging_v8i1e77140_app4.docx]

Multimedia Appendix 4


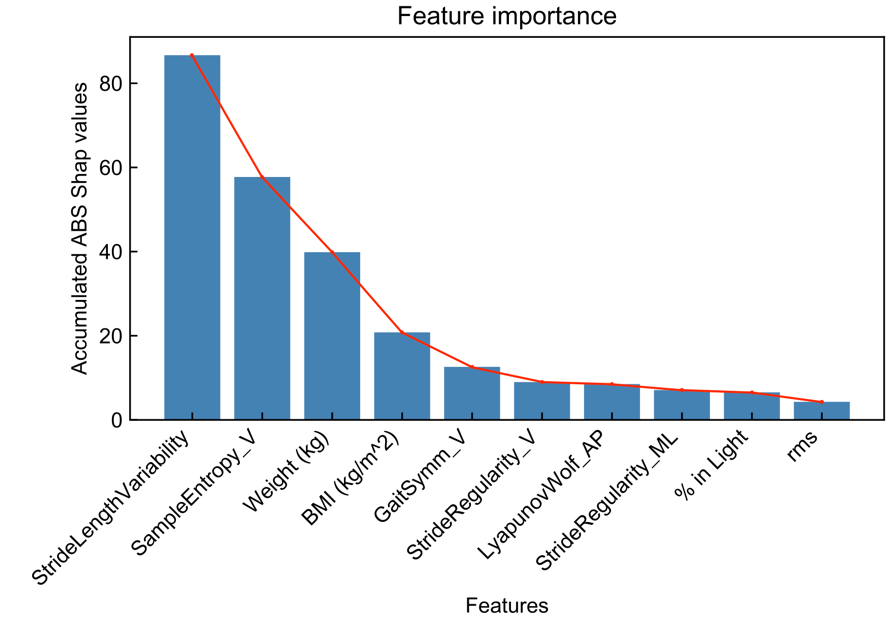


Supplement Figure 1. Absolute SHAP value for Top 10 outcomes and elbow plot. AP: anterior-posterior direction, BMI: body mass index, rms: Root mean square of acceleration signals, GaitSymm: gait symmetry index, V: vertical direction.
