# Supplementary material for: Machine Learning Approach for Frailty Detection in Long-Term Care Using Accelerometer-Measured Gait and Daily Physical Activity: Model Development and Validation Study
Source: JMIR Aging. 2025 Sep 15;8:e77140. doi: 10.2196/77140 (PMC12481141; doi:10.2196/77140)
Supplement: Multimedia Appendix 5 [file aging_v8i1e77140_app5.docx]

Multimedia Appendix 5

In this study, the gait symmetry score, as refer to Tura et al. [1], is calculated using the following formula:

$$Gait symmetry \mathrm{score}=1-\frac{{|Ad}_{1}-{Ad}_{2}|}{mean({Ad}_{1}+{Ad}_{2})}$$

where $Ad$ represents the peak of the autocorrelation of the acceleration signal.

[1] Tura, Andrea, et al. "Gait symmetry and regularity in transfemoral amputees assessed by trunk accelerations." Journal of neuroengineering and rehabilitation 7.1 (2010): 4.
