# Supplementary material for: Machine Learning Approach for Frailty Detection in Long-Term Care Using Accelerometer-Measured Gait and Daily Physical Activity: Model Development and Validation Study
Source: JMIR Aging. 2025 Sep 15;8:e77140. doi: 10.2196/77140 (PMC12481141; doi:10.2196/77140)
Supplement: Multimedia Appendix 6 [file aging_v8i1e77140_app6.docx]

Multimedia Appendix 6

| 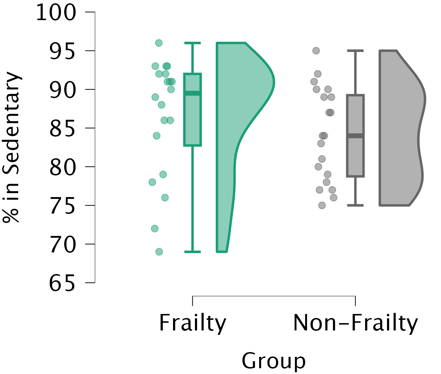 | 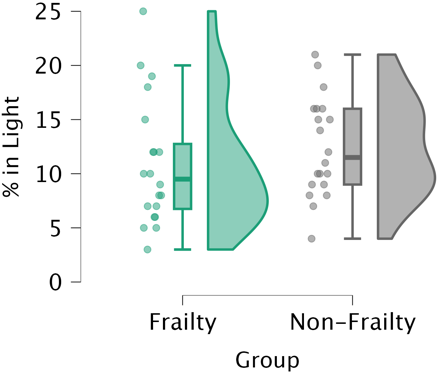 | 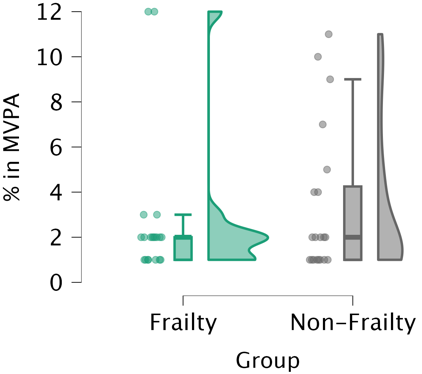 |
| --- | --- | --- |
| (a) Percentage of time spent in sedentary | (b) Percentage of time spent in light physical activity | (c) Percentage of time spent in moderate-vigorous physical activity |

Supplement Figure 2. Raincloud plot for the daily physical activity outcomes.
