# Supplementary material for: Machine Learning Approach for Frailty Detection in Long-Term Care Using Accelerometer-Measured Gait and Daily Physical Activity: Model Development and Validation Study
Source: JMIR Aging. 2025 Sep 15;8:e77140. doi: 10.2196/77140 (PMC12481141; doi:10.2196/77140)
Supplement: Multimedia Appendix 7 [file aging_v8i1e77140_app7.docx]

Multimedia Appendix 7


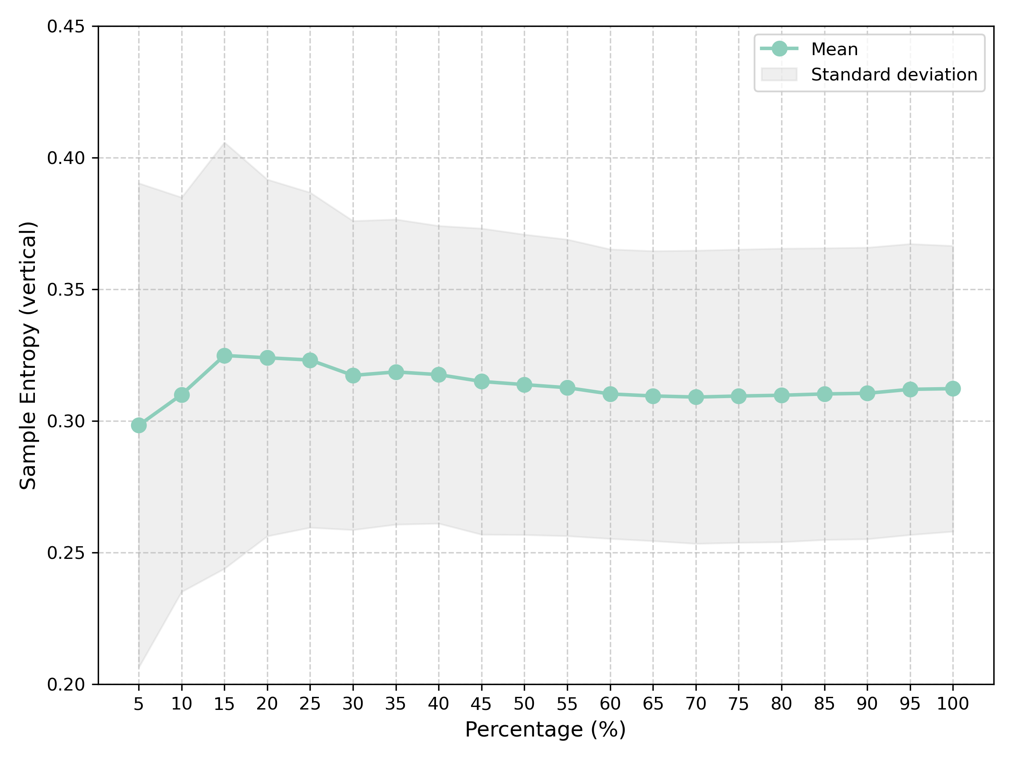


Supplement Figure 3. The averaged sample entropy values in vertical direction over all participants based on different length of acceleration signals.
